# 驱动基因阳性非小细胞肺癌免疫治疗进展

**DOI:** 10.3779/j.issn.1009-3419.2022.102.06

**Published:** 2022-03-20

**Authors:** 仁芳 邓, 月 曾, 越 潘, 春宏 胡, 芳 吴

**Affiliations:** 1 410011 长沙，中南大学湘雅二医院肿瘤中心 Department of Oncology, The Second Xiangya Hospital, Central South University, Changsha 410011, China; 2 412000 株洲，株洲市二医院肿瘤一科 Department of Oncology, Zhuzhou Second Hospital, Zhuzhou 412000, China; 3 410011 长沙，肿瘤大数据智能化应用湖南省工程研究中心 Hunan Cancer Mega-data Intelligent Application and Engineering Research Centre, Changsha 410011, China; 4 410011 长沙，肿瘤模型与个体化诊治研究湖南省重点实验室 Hunan Key Laboratory of Tumor Models and Individualized Medicine, Changsha 410011, China; 5 410011 长沙，肺癌早期诊断与精准治疗湖南省重点实验室 Hunan Key Laboratory of Early Diagnosis and Precision Therapy in Lung Cancer, Changsha 410011, China

**Keywords:** 肺肿瘤, 驱动基因阳性, 免疫治疗, Lung neoplasms, Immune therapy, Driver gene

## Abstract

肺癌是目前世界上致死率最高的恶性肿瘤，其中约80%为非小细胞肺癌（non-small cell lung cancer, NSCLC）。大部分NSCLC患者伴有“驱动基因突变”，针对突变基因的靶向治疗可取得较好的疗效，但仍有部分患者在治疗后会出现进展或复发，预后较差。已有的研究表明，免疫检查点抑制剂可改善晚期NSCLC的预后，延长患者生存期。但对于具有不同免疫微环境和分子特性的NSCLC患者，免疫治疗的效果差异较大。其中免疫治疗在驱动基因阳性的NSCLC患者中的地位存在比较大的争议。本文对驱动基因阳性NSCLC的免疫特点及免疫治疗在驱动基因阳性患者中的应用前景和挑战进行综述。

## Abstract (en)

Lung cancer is the most lethal malignancy around the world and non-small cell lung cancer (NSCLC) accounts for 80% of all cases. Most of the NSCLC patients has "driver gene mutations" and targeted therapy achieved a relatively good efficacy, but some patients progressed or relapsed after treatment. Previous studies demonstrated that immune checkpoint inhibitor could improve the prognosis of advanced-stage NSCLC and prolong the survival time. However, the efficacy of immune therapy varies in NSCLC patients with different immune and molecular features. The efficacy of immune therapy was controversial in NSCLC patients with driver gene mutation. The present review will summarize the immune characteristics of NSCLC patients with driver mutation and the directions of immunotherapy for patients with driver mutation.

肺癌是目前世界上致死率最高的恶性肿瘤，根据肿瘤细胞的形态学特征，肺癌分可为小细胞肺癌和非小细胞肺癌（non-small cell lung cancer, NSCLC），其中NSCLC占80%-85%^[1-2]^。分子病理学^[3-5]^证实，约73.9%的NSCLC患者有“驱动基因突变”，常见突变包括表皮生长因子受体（epidermal growth factor receptor, *EGFR*）、间变性淋巴瘤激酶（anaplastic lymphoma kinase, *ALK*）和*c-ros*原癌基因1酪氨酸激酶（c-ros oncogene 1 receptor kinase, *ROS1*）等。随着针对特异性靶点药物的应用，晚期NSCLC的5年生存率已提高至21%，而局限期患者约为55%^[6]^。虽然靶向治疗药物可显著改善驱动基因阳性NSCLC患者的预后^[6]^，但靶向药物耐药后，部分患者仍需要接受传统的含铂类药物的联合化疗。

免疫治疗是一种新兴的抗肿瘤治疗手段，可改善晚期NSCLC患者的生存，然而，仅约20%的NSCLC患者能从该治疗中获益^[7]^。目前，驱动基因阳性NSCLC患者能否从免疫治疗中获益是热点问题之一。NSCLC中的驱动基因与程序性细胞死亡蛋白1（programmed cell death protein 1, PD-1）/程序性细胞死亡配体（programmed cell death ligand 1, PD-L1）信号通路存在相互作用关系。研究数据^[8-11]^显示，免疫治疗可使部分驱动基因阳性NSCLC患者获益，提高患者的缓解率，并延长生存期。本文将对免疫治疗在驱动基因阳性NSCLC患者中的应用进展进行综述。

## 1 免疫检查点抑制剂在*EGFR*突变NSCLC患者中的应用

### 1.1 *EGFR*突变与PD-L1表达的关系

EGFR是上皮生长因子（epidermal growth factor, EGF）细胞增殖和信号传导的受体。NSCLC中EGFR通路可以调控PD-L1的表达。在NSCLC细胞中，增加EGFR的激酶活性可以激活下游的相关通路，并促进肿瘤的发生。EGFR可通过MAPK/p-ERK1/2、ePI3K/Akt/mTOR和IL-6/JAK/STAT通路来影响肿瘤发生发展^[12]^。研究^[13]^表明，EGFR通路可通过调控p-ERK1/2p-c-Jun信号轴来促进肿瘤细胞PD-1和PD-L1的表达，进而介导肿瘤的免疫逃逸，促进肿瘤发展。Azuma等^[14]^对164个NSCLC患者术后组织标本进行免疫组化分析，结果显示，*EGFR*突变与组织PD-L1高表达显著相关，*EGFR*突变是调控PD-L1蛋白表达的一个独立因素。可见，在NSCLC中，*EGFR*突变可以上调PD-L1的表达。

### 1.2 免疫治疗在*EGFR*突变NSCLC患者中的疗效

在一项回顾性研究^[15]^中，PD-1抑制剂在PD-L1高表达的*EGFR*突变NSCLC患者中可取得较好的疗效，17例有*EGFR*突变且伴有PD-L1高表达的患者在接受PD-1抑制剂治疗后客观缓解率（objective response rate, ORR）为29.4%，中位总生存期（overall survival, OS）达到26.4个月。但是，大部分临床研究亚组分析结果显示，单药免疫治疗*EGFR*突变NSCLC的效果并不理想。CheckMate012研究^[16]^显示，纳武利尤单抗治疗的*EGFR*突变患者生存期明显低于*EGFR*未突变患者[中位无进展生存期（median progression-free survival, mPFS）：8.8个月*vs* 1.8个月；中位总生存期（median overall survival, mOS）：未达到*vs* 18.8个月]。在KEYNOTE-001的研究^[17]^中，使用帕博利珠单抗（K药）治疗26例EGFR酪氨酸激酶抑制剂（tyrosine kinase inhibitor, TKI）耐药的NSCLC患者的ORR仅为4%，中位OS为120 d。

对于*EGFR*突变的NSCLC患者，免疫单药治疗的疗效有限，使得该部分患者在临床上较少采用免疫治疗，临床上也并不推荐对驱动基因阳性患者首选免疫治疗。但是，有研究结果显示，免疫联合治疗在*EGFR*突变NSCLC中可取得较好的效果。IMpower150研究^[8]^是一项纳入了1, 202例NSCLC患者的多中心III期临床试验，共分为3个亚组：（A）ACP组：阿替利珠单抗+卡铂+紫杉醇；（B）BCP组：贝伐珠单抗+卡铂+紫杉醇；（C）ABCP组：阿替利珠单抗+贝伐珠单抗+卡铂+紫杉醇。在伴有*EGFR*突变的患者中，ABCP组的中位OS明显高于BCP组（29.4个月*vs* 18.1个月，HR=0.6）。在一项II期临床试验^[18]^中，40例*EGFR*突变的NSCLC患者在出现靶向药耐药之后，接受阿特珠单抗联合贝伐珠单抗、培美曲塞和卡铂治疗后疾病控制率达到100%，患者的mPFS为9.4个月，1年OS率为72.5%，mOS尚未达到。免疫治疗联合EGFR靶向药物在NSCLC的治疗作用亦有报道。TATTON评估了PD-L1抑制剂Durvalumab联合奥希替尼在*EGFR*突变NSCLC患者中的效果^[19]^。结果显示，TKI初治患者（11例）的ORR为70%；而在23例TKI经治NSCLC患者中，T790M阳性突变患者的ORR达到67%，而T790M未突变患者的ORR仅为21%。一项开放标签、多中心、多队列、Ib期/II期研究^[20]^显示，对于*EGFR*或*ALK*驱动基因突变的复发或转移性非鳞NSCLC患者，卡瑞利珠单抗联合阿帕替尼展现出一定的临床获益，ORR为18.6%，1年OS率达到57.2%。在2020美国临床肿瘤学会（American Society of ClinicalOncology, ASCO）上，国内首个针对中国*EGFR*突变患者的前瞻性CT18研究，即特瑞普利单抗联合化疗用于EGFR-TKI治疗失败的*EGFR*突变阳性T790M阴性晚期NSCLC患者II期研究进行了相关报道，ORR达到50.0%，总体人群PFS达7.0个月^[21]^。目前，一项在TKI耐药的*EGFR*突变型转移性非鳞状细胞NSCLC受试者中进行的关于培美曲塞+铂类化疗联合或不联合帕博利珠单抗（MK-3475）的随机、双盲、III期研究（KEYNOTE-789）临床研究仍处于进行之中，结果尚未公布。

总之，*EGFR*突变患者对免疫检查点抑制剂单药治疗的反应较差，而免疫联合治疗方案可取得较好的疗效，且患者耐受性较好，这种联合治疗的方法为*EGFR*突变患者的治疗带来了曙光，也是未来的研究方向之一。

## 2 免疫检查点抑制剂在*ALK*阳性NSCLC患者中的应用

### 2.1 *ALK*融合与PD-L1的关系

*ALK*也是NSCLC患者一个重要的驱动基因，阳性率为3%-7%。有研究^[14]^报道，棘皮动物微管相关蛋白（echinoder mmicrotubule associated protein-like 4, *EML4*）-*ALK*阳性的肿瘤细胞中PD-L1表达水平较高，且*EML4*-*ALK*融合蛋白可上调肿瘤细胞中PD-L1的表达，而调低EML4-ALK后PD-L1的表达有所下降，说明NSCLC中该融合蛋白可调控PD-L1的表达水平。

### 2.2 免疫治疗在*ALK*融合NSCLC患者中的疗效

研究^[22]^显示，在伴有*ALK*融合的NSCLC患者中，如果PD-L1的阳性细胞表达率≥25%，免疫治疗也会有一定的效果。对于*ALK*融合的肺癌患者，在接受TKI治疗后，二线使用免疫治疗后的效果差于野生型的患者^[23, 24]^。但是，在有*ALK*融合的肺癌患者中，不论其PD-L1的表达情况，一线使用免疫治疗或使用TKI后二线使用免疫治疗的患者的OS明显低于使用化疗的患者^[10, 25]^。但随后的研究^[11]^显示，对于使用TKI药物后出现进展且伴有*ALK*融合的患者，二线使用阿特珠单抗联合贝伐珠单抗及铂类化疗药物，可延长患者的PFS。这些数据说明血管内皮生长因子抑制剂联合化疗具有免疫调控作用，可以增强免疫治疗的疗效，并提高这类患者的OS。

因此，对于有*ALK*融合的NSCLC患者，免疫治疗的效果有待进一步明确。但对于TKI治疗失败的NSCLC患者，免疫治疗联合化疗可能会延长患者的OS，使患者OS获益。

## 3 免疫检查点抑制剂在*KRAS*突变NSCLC患者中的应用

### 3.1 *KRAS*突变与PD-L1的关系

*KRAS*基因是NSCLC的另一个重要驱动基因，约20%的患者有*KRAS*基因突变，在吸烟的腺癌患者中更常见。*KRAS*基因突变可激活RAS-GTP酶，并影响肿瘤的增殖、迁移和血管生成等。既往的研究结果^[9, 26]^表明，NSCLC中*KRAS*突变可激活下游的相关通路并促进PD-L1表达，进而产生免疫逃逸，影响肿瘤发展。来自我国的一项大型回顾性研究^[27]^分析了NSCLC患者组织中基因突变状态与PD-L1表达的关系，发现*KRAS*突变患者中PD-L1表达阳性率为47.3%[肿瘤比例评分（tumor proportion score, TPS）≥1%]。Karatrasoglou等^[28]^研究亦证实NSCLC中*KRAS*突变与PD-L1表达呈正相关。可见，NSCLC中PD-L1的表达可受*KRAS*突变状态调控，且两者在组织层面上呈正相关。

### 3.2 免疫治疗在*KRAS*突变NSCLC患者中的疗效

目前，对于*KRAS*突变的患者，治疗上通常采用化疗联合免疫或单免疫治疗的方式，而*KRAS*基因状态可以影响NSCLC患者对免疫治疗的疗效。Passiglia等^[9]^研究结果显示，伴有*KRAS*基因突变的初治NSCLC患者可以从Nivolumab治疗中获益，但未突变的患者并没有获益。Amanam等^[29]^回顾性分析了60例NSCLC患者，其中大部分为IV期腺癌（87%），*KRAS*外显子12突变（78%），接受免疫疗法的患者（占20%）的mOS约为28个月，高于未接受免疫治疗的患者（33个月*vs* 22个月，*P*=0.31）。而Dong等^[26]^指出，*KRAS*突变状态与免疫治疗效果的相关性比较复杂。*KRAS*突变合并其他不同类型基因突变可能会影响免疫治疗疗效，*KRAS*突变常见的共存突变包括*TP53*、*STK11*。一项回顾性研究^[30]^分析了免疫治疗在174例*KRAS*突变肺腺癌患者中的效果，结论如下：如只有*KRAS*突变，患者对免疫治疗会有更好的效果；*KRAS*/*TP53*双突变的患者OS获益最大（ORR约为30%），而*KRAS*/*STK11*双突变的患者获益最少，提示*KRAS*/*TP53*共突变是指导免疫治疗的一个潜在预测指标。

从上述结果可以看出，NSCLC患者中*KRAS*基因与PD-1信号通路的关系比较复杂，目前大部分学者认为*KRAS*突变患者PD-L1表达更高，且*KRAS*突变患者较*KRAS*野生型NSCLC患者免疫治疗的效果可能更好，*KRAS*突变可能是NSCLC免疫治疗的一个重要预测指标，但目前仍缺乏大样本前瞻性多中心研究进行验证。

## 4 其他基因

其他相对少见基因包括*RET*、*BRAF*和*ROS1*等。*RET*基因主要编码一个受体酪氨酸激酶，该基因主要重排形成融合基因*KIF5B-RET*，*RET*重排在NSCLC中的发生率约为12%，是一个致瘤靶点^[31]^。在129例*RET*重排的NSCLC患者中，二代测序（next generation sequencing, NGS）发现41.1%（53/129）的患者伴有其他基因的改变，其中*TP53*突变最为常见（20/53, 37.7%），且PD-L1表达和肿瘤突变负荷（tumor mutation burden, TMB）水平与OS率无相关性，患者mPFS在化疗、免疫检查点抑制剂（immune checkpoint inhibitors, ICIs）和多靶点激酶抑制剂（multikinase inhibitors, MKIs）亚组之间无显著差异（mPFS：3.5个月、2.5个月和3.8个月），其中采用免疫抑制剂治疗的患者ORR仅为20%（2/10）^[32]^。

*BRAF*突变发生于1%-2%的NSCLC患者中，包括V600E和非V600E类型的突变。一项研究^[33]^回顾性分析了30例*BRAF*突变患者对免疫治疗的反应，其中A组为*BRAF* V600E突变（*n*=21），B组为*BRAF*非V600E突变（*n*=18），A组和B组的ORR分别为25%和33%，且PD-L1的表达水平与疗效之间无明显相关性。有研究^[34]^报道，TP53可增强PD-L1的表达水平，*TP53*突变的恶性肿瘤患者对免疫治疗的应答可能更好。*ROS1*融合基因是NSCLC患者一种相对罕见的基因突变，免疫治疗在*ROS1*融合阳性患者中的应用集中于个案报道^[35]^，其效果有待进一步研究。可见，免疫治疗在以上少见基因突变状态中的作用及机制目前仍不明确，未来需要更多的临床研究对其进行探索。

## 5 TMB与PD-L1及驱动基因突变之间的关系

TMB是预测免疫检查点抑制剂疗效的重要标志物之一。在NSCLC患者中，PD-L1表达和TMB的关系尚不完全肯定，既往的研究^[36, 37]^报道两者之间无相关性，但有研究^[38, 39]^表明两者之间存在一定的正相关。Yarchoan等^[38]^对9, 887例NSCLC患者组织样本进行了PD-L1和TMB检测，发现两者之间呈弱正相关。Lamberti等^[39]^研究纳入了421例NSCLC患者，使用NGS检测了组织标本中PD-L1的表达，发现TPS≥90%的患者有133例，而TPS < 1%的患者有288例，PD-L1高表达与TMB呈正相关（*P* < 0.001）。因此，NSCLC中TMB和PD-L1表达可能呈正相关，高表达TMB和PD-L1的患者在未来也许可以作为一个单独的亚群。

研究^[40]^发现，TMB在驱动基因阳性NSCLC患者中表达比较低。*EGFR*突变的NSCLC患者TMB相对较低，可能原因之一是*EGFR*突变的发生率在不吸烟患者人群中较高。另外，TMB在*EGFR*突变各亚型中也有差异，外显子21L858R突变患者中TMB明显高于外显子19缺失或20插入突变的患者^[41]^。尽管*ALK*突变与PD-L1表达没有什么关系，但其的确与低TMB密切相关^[42]^。目前关于NSCLC中驱动基因与TMB的关系报道较少，需要更多的前瞻性研究来进行验证。

## 6 驱动基因突变状态预测免疫治疗超进展

部分NSCLC患者在免疫治疗期间会出现超进展（hyperprogression, HP）现象。HP是指恶性肿瘤反常加速生长，包括免疫治疗后第一次评价时出现快速进展，肿瘤负荷相比基线增加 > 50%且肿瘤增长速度 > 2倍。免疫检查点抑制剂治疗后疾病HP的发生率为9%-29%^[43]^，这类患者预后极差，中位生存时间仅为2个月-5个月。研究^[44, 45]^表明，免疫治疗后发生HP的高危因素包括年龄、原发病灶的大小、肝脏或骨、CD39^+^CD8^+^ T细胞等。Kato等^[46]^发现，*EGFR*基因改变可能与免疫HP有关，10例*EGFR*改变且发生进展患者中有2例患者的进展速度分别增加了36倍和42倍。然而，Ferrara等^[47]^发现，16例*EGFR*突变患者均未发生免疫HP。因此，*EGFR*突变与免疫HP之间的关系尚不清楚。

## 7 免疫治疗联合治疗毒副作用分析

免疫治疗联合治疗在部分驱动基因阳性NSCLC患者中可取得较好的效果，但免疫治疗联合TKI抑制剂是否会增加毒副作用是需要关注的问题之一。免疫治疗联合EGFR-TKIs在肺癌中的毒副作用已有相关报道，免疫治疗联合靶向治疗并没有明显增加毒副作用，患者耐受性可，仅有少部分患者出现严重毒副反应^[20]^。在2020 ASCO上，国内首个针对中国*EGFR*突变患者的前瞻性CT18研究^[21]^，即特瑞普利单抗联合化疗用于EGFR-TKI治疗失败的*EGFR*突变阳性T790M阴性晚期NSCLC患者II期研究，仅15%的患者出现与化疗相关的恶心、呕吐、白细胞下降等不良反应。

## 8 总结与展望

近年来，以PD-1/PD-L1抑制剂为主的免疫治疗成为有希望治愈恶性肿瘤的治疗方法，部分驱动基因阳性的NSCLC患者能从免疫治疗中获益。对于*EGFR*突变的NSCLC患者，单药免疫治疗效果欠佳，但免疫治疗联合模式可以很大程度地改善该部分患者的预后。对于有*ALK*融合的NSCLC患者，免疫治疗联合化疗也有可能会延长患者的OS。而NSCLC患者中*KRAS*基因与PD-1信号通路的关系相对比较复杂，*KRAS*突变型患者免疫治疗的效果可能更好，伴随突变也会影响免疫治疗的疗效。免疫治疗联合化疗或联合抗血管生成靶向药物可提高其在驱动基因阳性NSCLC患者的有效率，延长患者的OS，且免疫联合化疗或靶向治疗并未明显增加毒副作用。

综上所述，精准治疗是未来NSCLC治疗的一个重要方向，而精确地筛选出免疫治疗如PD-1/PD-L1抑制剂的最大获益人群患者是我们面临的一个挑战。未来需要进行一系列多中心、前瞻性、大样本量的临床研究对免疫治疗疗效预测标志物进行探索。
